# Lessons learned from a diagnostic stewardship intervention in response to a blood culture bottle supply shock

**DOI:** 10.1017/ash.2025.10186

**Published:** 2025-10-06

**Authors:** Lior Cohen Yatziv, Alfredo Mena Lora, Alan E. Gross, Jenna Adams, Nahed Ismail, Scott Borgetti

**Affiliations:** 1 Division of Infectious Diseases, Department of Medicine, University of Illinois Chicago, Chicago, IL, USA; 2 Department of Pharmacy, UI Health, Chicago, IL, USA; 3 University of Illinois Chicago, Retzky College of Pharmacy, Chicago, IL, USA; 4 Invited Researcher, Chulalongkorn University, Bangkok, Thailand; 5 Department of Pathology; Clinical Microbiology, Laboratory Medicine, University of Illinois Chicago; University of Illinois Health System, Chicago, IL, USA

## Abstract

Faced with a national blood culture bottle shortage, our institution formed a multidisciplinary stewardship team and implemented policy changes. This reduced orders by 60%, potentially saving $145,000 monthly, while maintaining stable in-hospital sepsis mortality rates.

## Introduction

Healthcare systems worldwide have experienced recurrent supply shocks and shortages of critical medical equipment, underscoring the need for adaptable clinical practices and resource stewardship.^1^ Blood cultures (BCx) are a diagnostic cornerstone in sepsis management, enabling pathogen-directed therapy, reducing mortality, and improving clinical outcomes.^2^ Becton, Dickinson and Company (BD Diagnostics) notified the public of upcoming BCx bottle shortages in June 2024, prompting the United States Food and Drug Administration (FDA) and the Centers for Disease Control (CDC) to recommend mitigation strategies for healthcare providers, laboratory personnel, and healthcare facility administrators aimed at prioritizing the supply for patients at highest risk.^3^ Our facility implemented a diagnostic stewardship strategy aimed at restricting BCx ordering. We describe our BCx diagnostic stewardship intervention, as well as its impact on patient safety and clinical outcomes.

## Methods

This is a retrospective review of BCx utilization pre- and post-intervention, as well as sepsis outcomes at our institution. Ethical approval for this study was evaluated by UIC Institutional review board (IRB) and approved as IRB exempt.

### Study setting

The University of Illinois Hospital (UIH) is an urban, tertiary care hospital with 438 beds, including solid organ and bone marrow transplant services.

### Intervention

Drawing on diagnostic stewardship frameworks, a BCx stewardship algorithm categorized patients into three groups: (A) BCx not recommended, (B) two sets recommended, and (C) one set recommended for all other cases (see Supplement).^4^ The algorithm was shared via clinician emails, Electronic Medical Record (EMR) best practice alerts (BPAs), educational screensavers, flyers, and the institutional stewardship homepage. In-person education was led by the stewardship director and nurse educator, including reinforcement during audit-feedback rounds. Nurse-driven automatic BCx orders (eg, for sepsis alerts) were eliminated—only treating providers could order. Guidance discouraged repeating BCx within 48–72 hours unless clinically necessary.

### Data collection

We performed a retrospective review by retrieving EMR data of BCx orders, positivity, and contaminations per our microbiology Standard Operating Procedure (See Supplement) from January 2023 to January 2025 for patients age >18 years. Each BCx order included two bottles (single aerobic bottle and single anaerobic bottle). The preintervention period was January 1, 2023, to July 13, 2024. The postintervention period, excluding a 2-week implementation lead-in, was July 31, 2024, to January 1, 2025.

### Patient outcomes collection

Sepsis-related outcomes, including hospital sepsis prevalence and in-hospital mortality, are routinely collected for quality assurance and made available to clinical staff. We compared sepsis mortality rates before and after the intervention. Sepsis cases were defined as adult inpatients discharged with a final ICD-10 diagnosis of sepsis or septic shock. Mortality was defined as the proportion of these patients discharged deceased; ongoing admissions were excluded.

### Economic impact

Potential cost saving was estimated using a conservative direct cost of $96 per two-bottle set.^5^

We multiplied the total sets ordered by $96 and divided by the number of months to calculate the monthly reduction.

### Statistical analysis

Pre vs postintervention mortality was compared with a Yates-corrected χ^2^ test. We report the absolute risk difference (percentage points) with 95 % CI, χ^2^, and two-sided P. Analyses used R 4.3.3 (R Foundation).

## Results

The number of BCx, positivity, contamination, cost, and sepsis outcomes are depicted in Table 1.

**Table 1.** Positivity rate: calculated as positive cultures/bottles used. Contamination rate: calculated as contaminated bottles/bottles used. Cultures per 1,000 patient-days: calculated as (Bottles used/total days of admission) * 1,000. In-hospital sepsis rate: calculated as sepsis deaths/cases with sepsis diagnosis. Spendings ($): values are in US dollars

	Preintervention (1/1/23 – 7/14/24)	Postintervention (7/31/24 – 1/1/25)
Ordered blood culture sets	51,080	6,299
Positivity	3,611	545
Positivity rate	7.07%	8.65%
Contamination bottles	354	67
Contamination rate	0.69%	1.06%
Total days of admission	227.774	63.199
Cultures per 1.000 days	224.3	99.7
Spendings	$4,903,680	$604,704
Average cost per month	$265,064	$120,941
Sepsis diagnosis	1.694	591
Sepsis deaths	316	111
In-hospital sepsis rate	18.65%	18.78%

**Figure 1.**
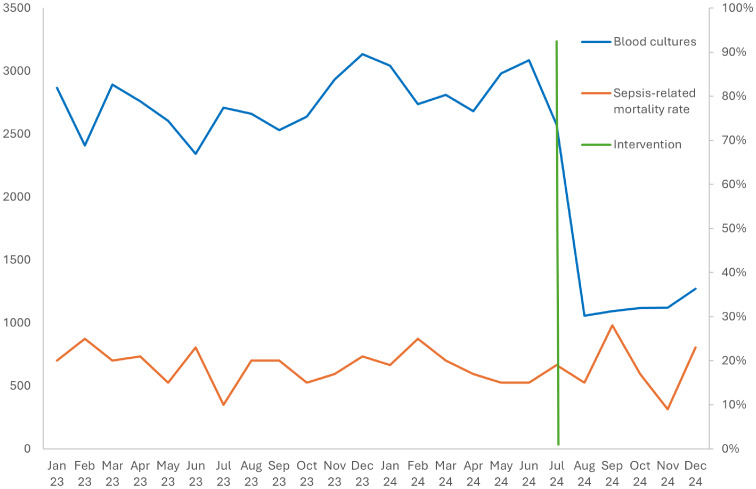
Blood culture orders per month and in-hospital sepsis mortality rate.

Preintervention, weekly blood-culture orders averaged 636 (224 per 1,000 patient-days). Postintervention, orders fell by 60 % to 259 per week (99 per 1,000 patient-days) (Figure 1). Mortality was 18.65 % (316/1694) in the preintervention period and 18.78 % (111/591) postintervention (risk difference = +0.13 pp; 95 % CI –3.5 pp to + 3.8 pp; χ^2^<.01; *P* = .99).

Estimated expenditures on bottles decreased by an average of $145,000 monthly.

## Discussion

The national BCx bottle shortage necessitated rapid adaptation at healthcare institutions nationwide.^6,7^ While the FDA and CDC^3^ provided general recommendations, specific strategies were left to individual institutions.

Our intervention was associated with a reduction in blood culture utilization while preserving patient safety. Key components of this strategy may be useful for other institutions seeking to achieve cost savings and prepare for future shortages.

The rapid formation of a multidisciplinary response team comprising clinicians, nurses, laboratory personnel, and IT staff allowed for the development and dissemination of institutional guidelines via meetings and digital platforms. An EMR alert was implemented, which provided clinicians with the relevant education when they were ordering BCx. Rather than a hard stop, this served as a technological checkpoint, prompting providers to reconsider the necessity of BCx in light of the ongoing shortage. By identifying common pitfalls, such as unnecessary BCx ordering and implementing BPAs in these areas, we can address broader healthcare challenges.^8^ Another key intervention was restricting BCx orders for patients who triggered an EMR sepsis alert, exclusively to the treating provider. This change promoted diagnostic stewardship by reinforcing clinical accountability, ensuring BCx were ordered intentionally based on clinical judgment rather than automated protocols.

The policy contributed to substantial reductions in utilization and cost, while diagnostic yield improved and contamination rate remained beneath the accepted ≤ 3 % threshold, reinforcing that fewer, more-targeted cultures can enhance clinical value without compromising quality.^5^ It raised provider awareness of different scenarios and the pretest probability of obtaining BCx. Our findings demonstrate that decreasing the rate of BCx ordering did not negatively impact in-hospital sepsis mortality, which was a major patient safety concern. Similar challenges have been faced by institutions nationwide, prompting the rapid deployment of various stewardship strategies and limitations on BCx use .^6^ The BCx bottle shortage led our facility and other institutions to rapidly adapt. As in prior unexpected supply shortages, a crisis can be an opportunity for learning and growth, ^9^ and what started as an acute need can potentially impact long-term practice. Diagnostic stewardship of BCx is a relatively new field of study, ^4^ and this event demonstrates the potential feasibility and impact of long-term BCx stewardship. Institutions should consider maintaining some of the implemented restrictions even after supply issues are resolved to prevent overutilization and reduce unnecessary spendings. Those changes can have even wider effects on overall costs due to potentially reduced nursing, lab processing time, and contamination rates.^4,5^ Furthermore, this intervention requires minimal resources to implement and suggests that similar successful stewardship models can be applied in future similar events.

### Limitations

This report has several limitations. As a single-center, retrospective observational analysis, it is subject to uncontrolled confounding and cannot establish causality. We did not capture detailed patient demographic or clinical variables and relied exclusively on ICD-10 sepsis codes, which may lead to misclassification. Granular data distinguishing single-set from multi-set BCx collections were unavailable, precluding a full assessment of adherence to the stewardship algorithm and its impact on positivity and contamination rates. Cost-savings calculations excluded indirect expenditures such as nursing and laboratory labor. Finally, this report observed a relatively brief period after the intervention, and further monitoring is warranted to validate these preliminary results.

### Conclusion

Our intervention was associated with weekly blood-culture orders reduction by 60%, lowering use from 224 to 99 cultures per 1,000 patient-days. The intervention was associated with an estimated monthly savings of ∼$145,000, and most notably, with no associated change in in-hospital sepsis mortality.

Further studies are warranted to validate this strategy and assess its long-term effectiveness and safety. Nevertheless, this intervention highlights how crises can catalyze meaningful change, driving operational efficiency, and more judicious resource allocation in healthcare while maintaining patient safety.

## Supporting information

10.1017/ash.2025.10186.sm001 Cohen Yatziv et al. supplementary material 1 Cohen Yatziv et al. supplementary material

10.1017/ash.2025.10186.sm002 Cohen Yatziv et al. supplementary material 2 Cohen Yatziv et al. supplementary material

## Data Availability

Data available upon reasonable request from the corresponding author.

## Supplementary material

The supplementary material for this article can be found at https://doi.org/10.1017/ash.2025.10186.
