# Supplementary material for: Lessons learned from a diagnostic stewardship intervention in response to a blood culture bottle supply shock
Source: Antimicrob Steward Healthc Epidemiol. 2025 Oct 6;5(1):e251. doi: 10.1017/ash.2025.10186 (PMC12509148; doi:10.1017/ash.2025.10186)

Supplementary material:

1. Criteria for calling a positive blood culture a Contaminant – Taken from Microbiology lab Standard Operating Procedure for processing and interpretation of positive blood cultures.

- 1. Isolate is identified as one of the following:
  2. microaerophilic Streptococcus and/or members of the Streptococcus viridans group. Streptococcus viridans group are not speciated. Sensitivity is performed only when the isolate is suspected not to be a contaminant. See criteria below for AST on these isolates.
  3. Corynebacterium species (diphtheroids)
  4. Bacillus species (not anthracis)
  5. Propionibacterium species
  6. coagulase-negative Staphylococcus

**AND**

1. Number of positive blood bottles:
   1. 1 out of 4 bottles
   2. 1 out of 2 bottles
   3. Blood culture stewardship algorithm that has been distributed to medical teams.


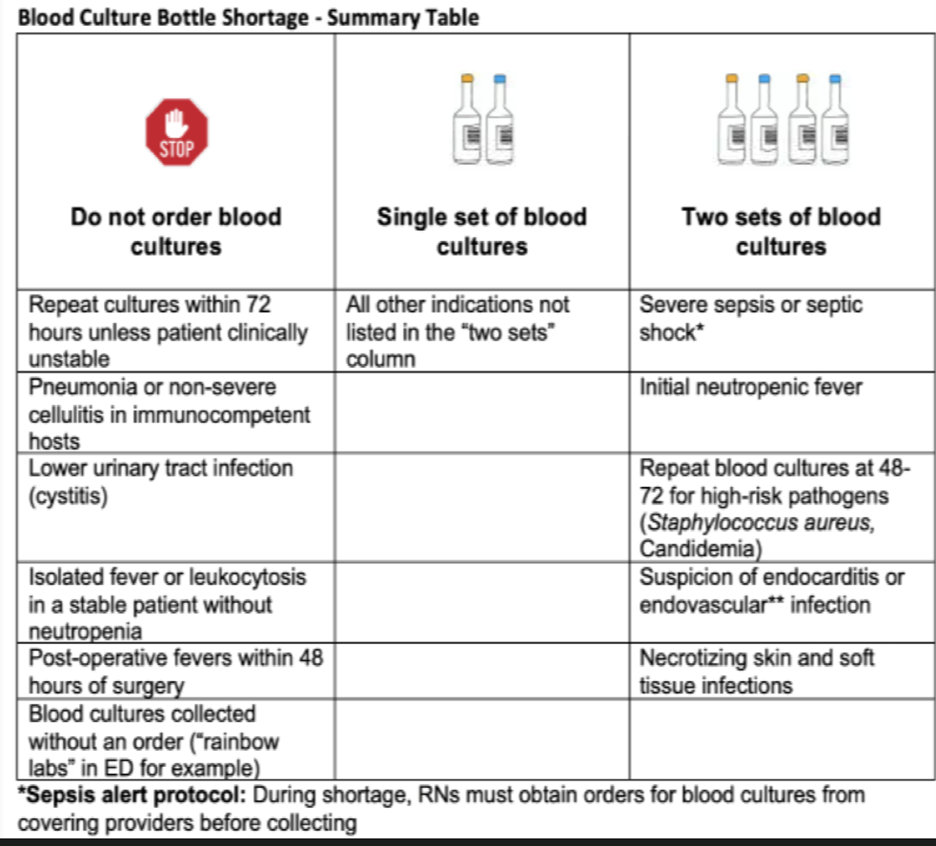

Supplement: Cohen Yatziv et al. supplementary material 1 — Cohen Yatziv et al. supplementary material [file S2732494X25101861sup001.docx]
